# Sistematic Reviews About Infertility and Robotic Surgery are the Hot Topics in this Issue of International Brazilian Journal of Urology

**DOI:** 10.1590/S1677-5538.IBJU.2025.04.01

**Published:** 2025-07-01

**Authors:** Luciano A. Favorito

**Affiliations:** 1 Universidade do Estado do Rio de Janeiro - Uerj Unidade de Pesquisa Urogenital Rio de Janeiro RJ Brasil Unidade de Pesquisa Urogenital - Universidade do Estado do Rio de Janeiro - Uerj, Rio de Janeiro, RJ, Brasil; 2 Hospital Federal da Lagoa Serviço de Urologia Rio de Janeiro RJ Brasil Serviço de Urologia, Hospital Federal da Lagoa, Rio de Janeiro, RJ, Brasil

The July-August 2025 number of *Int Braz J Urol* presents original contributions with a lot of interesting papers in different fields: Bladder dysfunction, Renal cell Carcinoma, Prostate Cancer, Robotic surgery, Telesurgery in Urology, Urolithiasis, Infertility and male sexuality. The papers came from many different countries such as Brazil, USA, Italy, Spain, Romania, India and China, and as usual the editor's comment highlights some of them. The editor in chief would like to highlight some papers in this number, specially the papers about the new visual erection hardness score:

Dr. Pires and collegues from Brazil, presented in id e20240614 (1) a nice systematic review about the efficacy of alpha lipoic acid (ALA) supplementation in sperm parameters and concluded that this meta-analysis comprehending five randomized control trials RCTs suggests strong evidence for ALA supplementation, which was associated with a favorable change in sperm function, especially spermatozoa motility. However, there were no effects on the pregnancy rates, likely due to the small sample size and short follow-up. Nevertheless ALA should be considered as an adjuvant treatment for patients with idiopathic infertility. Further prospective randomized controlled trials are needed, particularly those including pregnancy rates and longer follow-ups.

Dr. Andras and collegues from Romania and USA, presented in id e20250007 (2) a systematic review about the new Robotic Systems in Urology and concluded that the adoption of novel multi-port and single-port robotic systems in urologic surgery can offer new opportunities for enhanced precision, reduced invasiveness, and potentially improved patient outcomes. The variability in outcomes across different platforms underscores the need for continued research and standardized training.

Dr. Oliveira and collegues from Brazil, presented in id e20240567 (3) a nice randomized clinical trial about the darifenacin versus parasacral transcutaneous electric nerve stimulation (PTENS) for overactive bladder syndrome (OABSS) in patients infected with Human T-Lymphotropic Virus 1 and concluded that both protocols used in this study were effective in treating OAB syndrome and reducing OABSS. However, therapy abandonment and adverse events were more frequent in the darifenacin group compared to the PTENS group.

Dr. Jiao and collegues from China, presented in id e20240706 (4) a nice study about the clinical outcomes and cost-effectiveness between the Sentire® and da Vinci® systems in Robot-assisted Radical Prostatectomy and concluded that this study demonstrates that the Sentire® system is well-suited for urological surgeries, offering com- parable clinical outcomes and shorter hospital stays while improving cost-effectiveness compared to the da Vinci® system.

Dr. Li and collegues from China, presented in id e20250034, (5) a nice study about the risk-adjusted trifecta outcomes in ultrasound-guided RFA of T1a renal masses: experience from a large tertiary cancer center; this paper is the cover of this edition. The authors concluded that Ultrasound-guided RFA is a safe and effective treatment for T1a renal masses. Tumor size and the history of ipsilateral partial nephrectomy were significantly associated with trifecta failure.

Dr. Gentile and collegues from Brazil presented in id e20250039 (6) a nice study about the first step to equity and organization in waiting lines for stone surgery: Translation of Prioritization Scores and developed the Brazilian Portuguese version of the Western Canada Waiting List and SCQ-score. This development will allow further studies to evaluate the impact of their use within the Brazilian healthcare environment.

Dr. Moschovas and collegues from USA presented in id e20250083 (7) a nice study about exploring the teleproctoring potential of telesurgery: The rirst remote procedures performed simultaneously between Orlando and Shanghai and concluded that this study demonstrates the feasibility of long-distance telesurgery and high- lights its potential to improve surgical outcomes, facilitate training, and offer remote assis- tance for complex cases. Telesurgery could play a significant role in expanding access to specialized care and enhancing robotic surgical training globally.

The Editor-in-chief expects everyone to enjoy reading.
